# The endophytic fungus *Penicillium oxalicum* isolated from *Ligusticum chuanxiong* Hort possesses DNA damage-protecting potential and increases stress resistance properties in *Caenorhabditis elegans*

**DOI:** 10.3389/fphar.2022.983716

**Published:** 2022-08-30

**Authors:** Zizhong Tang, Yihan Qin, Yueyu Wang, Wenjie Lin, Qing Wang, Nayu Shen, Yirong Xiao, Hong Chen, Hui Chen, Tongliang Bu, Qingfeng Li, Huipeng Yao, Shiling Feng, Chunbang Ding

**Affiliations:** ^1^ College of Life Sciences, Sichuan Agricultural University, Ya’an, China; ^2^ Sichuan Agricultural University Hospital, Sichuan Agricultural University, Ya’an, China; ^3^ College of Food Science, Sichuan Agricultural University, Ya’an, China

**Keywords:** endophytic fungi, *Penicillium oxalicum*, antioxidant, oxidative stress resistant, *Caenorhabditis elegans*, DNA damage protection

## Abstract

The chemical composition and antioxidant activity of extracts (POE) of *Penicillium oxalate* isolated from *Ligusticum chuanxiong* Hort have been investigated. However, the biological activity of POE is limited, and its antioxidant, stress resistance and DNA protection effects *in vivo* are unclear. The current study aims to explore the beneficial effects of POE on DNA damage protection in pBR322 plasmid and lymphocytes and stress resistance in *Caenorhabditis elegans*. The results showed that POE increased the survival rate of *C. elegans* under 35°C, UV and H_2_O_2_ stress, attenuated ROS and MDA accumulation, and enhanced the activity of some important enzymes (SOD, CTA, and GSH-PX). In addition, the POE-mediated stress resistance involved the upregulation of the expression of the *sod-3*, *sod-5*, *gst-4*, *ctl-1*, *ctl-2*, *daf-16*, *hsp-16.1*, *hsp-16.2*, and *hsf-1* genes and acted dependently on *daf-16* and *hsf-1* rather than *skn-1*. Moreover, POE also reduced lipofuscin levels, but did not prolong the lifespan or damage the growth, reproduction and locomotion of *C. elegans*. Furthermore, POE showed a protective effect against DNA scission in the pBR322 plasmid and lymphocytes. These results suggested that *P. oxalate* extracts have significant anti-stress and DNA protection potential and could be potential drug candidates in the pharmaceutical field, thus greatly broadening the understanding of the biological effects of the endophytic fungus *P. oxalate*.

## 1 Introduction

There is increasing evidence that reactive oxygen species (ROS, e. g., O_2_− and OH) and free radical-meditated reactions damage DNA, lipids and proteins (Dubois et al., 2018), eventually leading to various diseases. For example, DNA damage affects normal physiological metabolism and blocks some metabolic pathways, leading to ageing, cancer, atherosclerosis, coronary heart ailment, diabetes, Alzheimer’s disease and other neurodegenerative disorders (Xu et al., 2005). Currently, antioxidants have various degrees of anti-inflammatory, antiatherosclerotic, antitumor, antimutagenic, anticarcinogenic, antibacterial or antiviral effects (Aruoma, 1998) and are considered highly effective in treating ROS-mediated pathologies. Many synthetic antioxidant compounds, such as butylated hydroxyanisole and butyl hydroxytoluene, are useful, but they are cytotoxic and are suspected to be potential causes of health damage (Conning and Phillips, 1986). Accordingly, finding efficient and safe antioxidants from natural resources to prevent and reduce the occurrence of related diseases is urgently warranted (Denis et al., 2013).

Currently, endophytic fungi isolated from medicinal plants have received greater attention due to their great potential to produce bioactive compounds with a variety of biological properties (Strobel, 2003; Strobel et al., 2004). Previous studies have suggested that endophytic fungus extracts contain bioactive substances with antibacterial, antioxidant and other bioactivities (da Silva et al., 2020), such as *Lasiodiplodia venezuelensis* isolated from *Syzygium samarangense* L (Budiono et al., 2019) and *Cercospora* sp. PM018 was isolated from *Lal-bisalyakarani* (Mookherjee et al., 2020), and could be a potential antioxidant resource for the treatment of related diseases. Moreover, *in vitro* fermentation culture of endophytic fungi has the advantages of high yield, a short fermentation period, high production efficiency, and sustainable production of target bioactive ingredients (Ludwig-Müller, 2015). Hence, the efficacy and potential usefulness of endophytic fungus extracts have led to a number of studies with the aim of detecting their antioxidant activity. However, there have been few studies on the oxidative stress resistance of endophytic fungus extracts *in vivo* models (Tiwari et al., 2014) and most studies have only confirmed the antioxidant activity of endophytic fungal extracts *in vitro* (Huang et al., 2007; Li et al., 2015). Fortunately, *C. elegans*, a powerful tool, is commonly used to test various physiological processes, the mechanisms of some diseases and the biological activity of natural products due to its advantages of small body size, ease of handling and many mutant strains (Wang et al., 2014). For instance, previous studies frequently used *C. elegans* as a model to explore the antioxidant, anti-stress and anti-ageing capacities of different plant extracts (Duangjan et al., 2021). In addition, as far as we know, DNA damage is associated with ROS imbalance, and excess free radicals can damage DNA strands leading to the occurrence of various diseases (Thanan et al., 2014). Nevertheless, an extensive survey of the literature revealed very few reports corroborating the protective potential against DNA damage of endophytic fungus extracts (Kaur et al., 2020).

In previous studies by our group, an extract of *Penicillium oxalate* (POE) isolated *Ligusticum chuanxiong* Hort was reported to have antioxidant capacity (Tang et al., 2021), but its antioxidant and oxidative stress resistance properties in animal models and DNA damage protection effects are lacking. Therefore, the purpose of this study was to enrich the biological effects of the endophytic fungus *Penicillium oxalicum*, such as anti-stress and DNA damage protection properties. Our present findings could accelerate the utilization of POE in the field of therapeutics by virtue of its DNA damage protection, antioxidant activity and increased stress resistance potential in *C. elegans*.

## 2 Materials and methods

### 2.1 Materials

The endophytic fungus *P. oxalate* was isolated from the roots of *L. chuanxiong* Hort and stored in the “Fermentation Engineering Laboratory” (College of Life Sciences, Sichuan Agricultural University, Ya’an, China).

The activated endophytic fungus *p. oxalate* was inoculated in PDA medium and cultured on a shaker for approximately 1 week at 28°C. Subsequently, vacuum-filtered fermentation broth was extracted with ethyl acetate and concentrated with a rotary evaporator to obtain the *P. oxalate* extract (POE).

### 2.2 *Caenorhabditis elegans* strains and culture conditions

The strains N2 (wild type), CF1038 [*daf-16* (mu86)I] (WBStrain00004840), EU1 [*skn-1* (zu67) IV/nT1 (IV; V)] (WBStrain00007249), PS3551 [*hsf-1* (sy441)I] (WBStrain00030901) and *Escherichia coli* OP50 (*E. coli* OP50) were obtained from the Caenorhabditis Genetics Center United States. Worms were cultured at 20°C in solid nematode growth medium (NGM) and seeded with inactivated *E. coli* OP50 as a food source. The worms were age synchronized based on the bleaching method as follows: eggs were obtained by bleaching adults using lysis solution 3.5 ml of M9 buffer, 0.5 ml of NaClO (5%) and 1 ml of NaOH (5 mol/L). Unless otherwise stated, all eggs were incubated on NGM plates containing *E. coli* OP50 and different concentrations of POE.

### 2.3 Acute toxicity assay

Toxicity tests in liquid medium were performed according to a previous method with modifications (Moliner et al., 2018). In brief, synchronized L4 worms were placed in M9 buffer in a 96-well microplate with different concentrations (1–100 μg/ml) of POE at 20°C for 24 h. At least 100 worms per condition were evaluated per treatment and M9 was used as a negative control. Subsequently, the survival rate (%) was calculated after 24 h.

Survival rate%=(Number of alive worms×100)/Total number of worms.

### 2.4 Stress resistance assay

Before exposure to the corresponding stressors, the age-synchronized L1 larvae worms were treated or not treated with POE (25, 50, 75 µg/ml) for 3 days at 20°C. Subsequently, the late L4 larvae or young adult were washed twice with sterile water and exposed to various stresses until all individuals died. The worms were considered dead when they did not respond to platinum wire stimulus. All trials were repeated three times. Resveratrol (Res, 22.5 µg/ml) was used as a positive control (Zhuang et al., 2016).

#### 2.4.1 Ultraviolet-B stress assay

To evaluate resistance to UV irradiation, the POE-treated worms were exposed to UV irradiation (120 mJ cm^−2^) for 4 h. The number of surviving worms was counted every 24 h (Wang et al., 2018).

#### 2.4.2 H_2_O_2_-induced oxidative stress assay

This assay was performed as described previously (Saul et al., 2008). Briefly, the POE-treated worms were transferred to fresh NGM containing 2 mM H_2_O_2_ to determine the effects of POE on oxidative stress. The survival rate of the worms was observed every 30 min.

#### 2.4.3 Heat shock assay

The heat shock assay using *C. elegans* was performed according to Lin et al. (2019). The POE-treated worms were moved from a comfortable cultivation environment (20°C) to a 35°C mediated stress environment. Subsequently, the number of surviving worms was monitored every hour to determine their heat stress resistance.

### 2.5 Intracellular malondialdehyde content, and superoxide dismutase, catalase, and glutathione peroxidase activities

The POE (25, 50, 75 µg/ml)-treated worms (L4 stage) were treated with and without H_2_O_2_ (2 mM) for 1 h. Next, worm bodies were lysed by ultrasound equipment and supernatant was obtained after centrifugation. The MDA and protein content, SOD, CAT, and GSH-Px activity were determined according to the commercial assay kits (Nanjing Jiancheng Biotechnology Institute, China). Final results were normalized to protein levels (Xiao et al., 2014).

### 2.6 Reactive oxygen species accumulation assay

Estimation of endogenous ROS levels was based on the method described by Prasanth et al. (2019). Briefly, the worms were treated with different concentrations of POE (25.50 and 75 μg/ml) for 3 days and exposed to oxidative stress (2 mM H_2_O_2_) for 1 h. Then, the worms were washed thoroughly with M9 buffer and incubated with 5 μg/ml 2′,7′-dichloro-fluorescein diacetate (DCFH-DA) for 20 min, followed by another wash to remove the excess DCFH-DA. Furthermore, the worms were transferred with a drop of sodium azide (0.5%) onto a glass slide. Fluorescent imaging was performed on 10 worms using an Olympus FV1200 confocal microscope (Tianjin Leike Optical Instruments Co., Ltd.). The relative fluorescence was measured and calculated using ImageJ software.

### 2.7 Lipofuscin accumulation and body length assay

The lipofuscin level was measured after 5 days of POE treatment. Then the worms were randomly selected and washed with M9 buffer three times and then anesthetized with 0.5% NaN_3_ as described in previous study (Onken and Driscoll, 2010). At least 10 worms were selected for imaging using a fluorescence microscope (CX23, Olympus, Tokyo, Japan) at wavelength with excitation/emission (360/420 nm) filters. The fluorescence intensity and the body size of the worms were measured using ImageJ software.

### 2.8 Longevity assay

The N2 worms were used for lifespan analysis under normal conditions as described in previous study (Duangjan et al., 2019a). In brief, synchronized L4 larval worms were placed on NGM plates with POE. Then, live worms were counted and transferred to fresh NGM plates containing POE every day until all individuals died. The L4 worms were defined as a starting time point (d 0) for lifespan assay. The assay was performed with approximately 100 worms in each group and the results are expressed as the survival rate%.

### 2.9 Fertility assay

The fertility assay was performed as described in a previous study (Lin et al., 2020). In brief, reproductive capacity was evaluated by three indexes: brood size, progeny number and hatchability (the ratio of progeny number to brood size number). The parents of the worms were transferred daily to fresh NGM containing 50 μg/ml POE during the progeny production period. Then, the eggs on the old NGM were counted daily. Moreover, the old NGM was kept at 20°C for 24 h to detect viable eggs. The experiment was performed with at least 10 worms per group.

### 2.10 Movement assays

The body movement assay was performed as described previously (Herndon et al., 2002). The age-synchronized L1 larvae worms were treated and the motility of worms was evaluated on Days 3, 7, and 10. Then, worms were transferred to fresh plates for 1-min of free movement. Subsequently, the motility behaviour of worms was observed using a stereomicroscope and was classified into classes A, B, and C: the highly mobile worms, which we designated class A, moved spontaneously and smoothly; members of class B did not move unless prodded, and they left tracks that were nonsinusoidal; and class C worms did not move forward or backwards, but oscillated their nose or tails in response to touch.

### 2.11 Expression levels of gene assays

The worms were treated with or without 50 µg/ml POE for 72 h from eggs and then incubated with 2 mM H_2_O_2_ for 1 h. Total RNA was extracted using the TRIzol Total RNA Extraction Kit (Tiangen, Beijing, China) and synthesized into cDNA using the FastKing RT Kit (TSINGKE Biotech Co., Ltd., Beijing, China). Subsequently, quantitative reverse transcription polymerase chain reaction (qRT‒PCR) was performed using SuperReal PreMix Plus (SYBR Green) and a real-time PCR detection system (Bio-Rad, Laboratories, Hercules, CA, United States). The expression of mRNA was analysed using the comparative 2^−ΔΔCt^ method and *act-1* was the internal control gene. The primers used for qRT‒PCR in this study are listed in Supplementary Table S1.

### 2.12 Determination of DNA damage protective activity

#### 2.12.1 DNA nicking assay for hydroxyl radical scavenging activity

The potential of POE to protect the supercoiled pBR322 plasmid from the destructive effect of free radicals caused by the Fenton reagent was estimated using the DNA nicking assay as described by Jeong et al.(2009). Five microlitres of PBS (10 mM), 2 µl of plasmid DNA (0.5 µg), POE (5 µl, 25, 50, and 75 μg/ml), 2 µl of FeSO_4_(1 mM) and 2 µl of H_2_O_2_(1 mM) were mixed. The reaction mixture was incubated for 30 min at 37°C. After incubation, 2 µl of loading buffer were added to stop the reaction and the DNA was analysed with 1% agarose gel electrophoresis for 30 min under 120 V. Subsequently, the different forms of DNA, i.e., Supercoiled (SC) and open circular (OC) DNA were visualized and semi-quantitative analysis to calculate the double helix percentage under the gel documentation system (Gel Doc XR, Bio-Rad, United States). The positive control was 500 µM vitamin E (VE) (Liu et al., 2022).

$$\text{Double helix rate}\;\left(\%\right)={\text{A}}_{0}/\left({\text{A}}_{0}+{\text{A}}_{1}\right)\times 100\%$$

where *A*_0_ is the grey value of the double helix conformation, and *A*_1_ is the grey value of the open-loop conformation.

#### 2.12.2 Cytochalasin blocked micronucleus assay in lymphocytes

In this assay, lymphocytes were cultured by adding 500 µl of whole blood with 9 ml of RPM11640, 10% foetal bovine serum, penicillin (100 units/ml), streptomycin (100 µg/ml) and phytohemagglutinin (5 µg/ml). Then, the cells were exposed to H_2_O_2_ (250 µM) to induce DNA damage. Simultaneously, POE at different concentrations (25 50 and 75 μg/ml) was added to the cultures for 72 h in 5% CO_2_ at 37°C. At 44 h, cytochalasin-B (3 µg/ml) was added to the cultures to block cytokinesis. At 72 h, the cultures were collected and treated (Carvalho-Silva et al., 2016). Coded slides were stained for 30 min with Giemsa and observed under a microscope. MN and other nuclear abnormalities were scored in 500 well spread cells of each culture (Fenech et al., 1999).

### 2.13 Statistical analysis

Survival curves were drawn to determine significant differences using log-rank (Mantel-Cox) tests (GraphPad Software, CA, United States) (*p < 0.05, **p < 0.01, and ***p < 0.001). Other statistical calculations used one-way ANOVA followed by LSD and Duncan tests (SPSS software, version 20). All data are expressed as the mean ± SD (*n* = 3), and different letters in columns indicate that the values are significantly different (*р* < 0.05).

## 3 Results

### 3.1 Effect of *P. oxalate* extract on acute toxicity in *C. elegans*

The acute toxicity of POE was initially assessed by studying the effect of POE on the viability of *C. elegans*. The results showed that, compared with the control group, the viability of the worms was not affected after 24 h of POE treatment at concentrations ranging from 10 to 100 µg/ml (Table 1). Worms exposed to maximum dose extracts also maintained a survival rate of 93% ± 4%, while the viability rates of the control group were 92% ± 1% (Table 1). Thus, POE did not produce acute toxicity to the model organism at the tested concentrations.

**TABLE 1:** Effects of POE on the viability of *C. elegans*: The results are presented as mean of viability ± SEM%.

Concentration (µg/ml)	Survival rate (%)	*p*-value
Control	92 ± 1	
100	93 ± 4	>0.05
80	94 ± 5	>0.05
60	95 ± 3	>0.05
40	93 ± 3	>0.05
20	91 ± 4	>0.05
10	93 ± 5	>0.05

Not significance differences between treatment and control groups found (*p* > 0.05).

### 3.2 Effect of *P. oxalate* extract on stress in *C. elegans*

To comprehensively evaluate the stress resistance of POE, we measured the lifespan of worms under conditions of ultraviolet radiation, H_2_O_2_ and 35°C.

**FIGURE 1:**
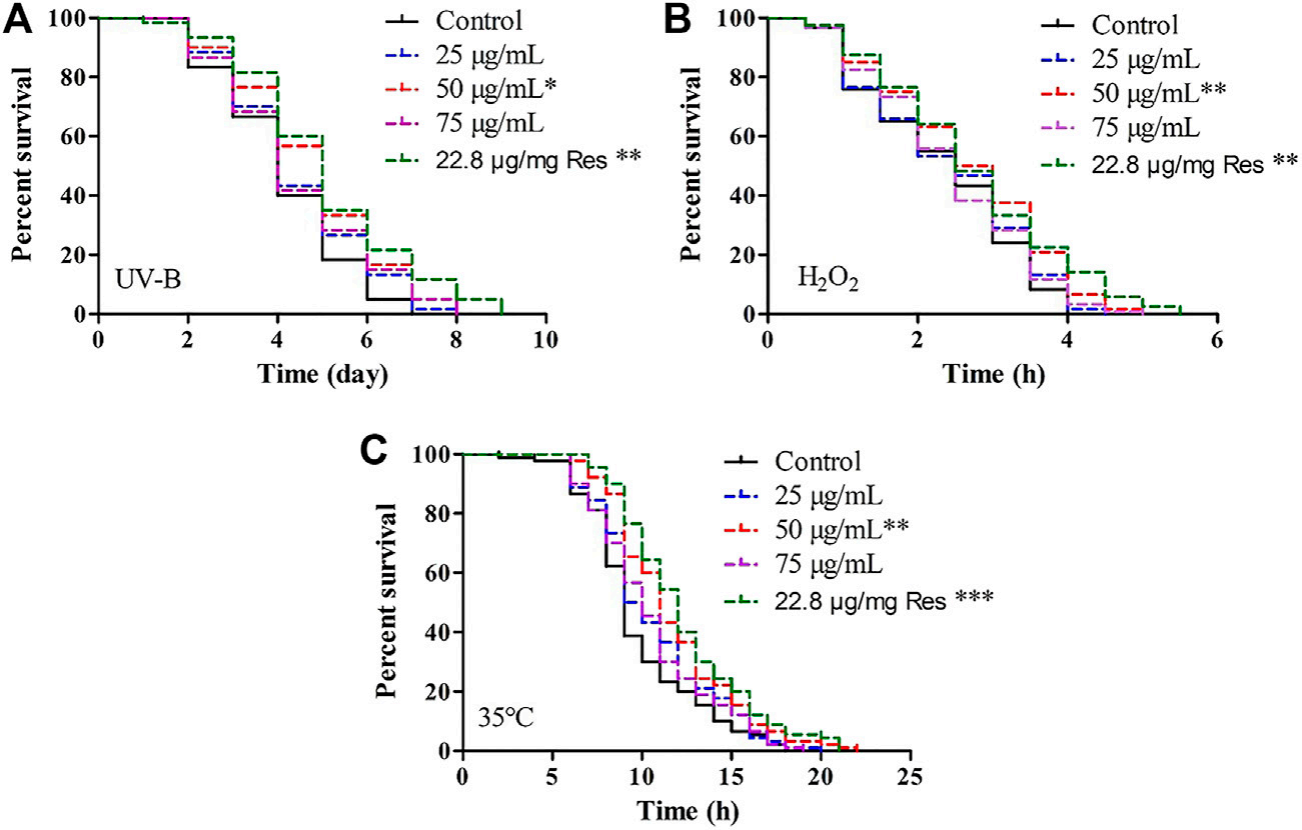
The effect of POE on stress resistance in *C. elegans*. **(A)** Survival curve of worms under UV irradiation-induced stress. **(B)** Survival curve of worms under H_2_O_2_-induced stress. **(C)** Survival curve of worms under 35°C-induced stress. Three independent biological replicates were performed. Differences compared to control group were considered significant at *p* < 0.05 (*), *p* < 0.01 (**) and *p* < 0.001 (***).

First, we found that the treatment in the presence of POE (50 μg/ml) promoted right shift in the worm survival curve under UV radiation when compared with the controls (Figure 1A), and the mean lifespan of worms treated with UV radiation was increased by 6.4%, 14.0%, and 6.9% in the 25, 50, and 75 μg/ml treatment groups, respectively, compared with the control group (Supplementary Table S2), although the difference was only significant in the group treated with 50 µg/ml POE (Supplementary Table S2; *p* < 0.05). Furthermore, there was no significant difference in the maximum lifespan between the treatment group (50 μg/ml) and the control group, but the mean and median lifespans were increased significantly (Supplementary Table S2; *p* < 0.05). Second, in the H_2_O_2_-induced oxidative stress assay, a similar protective effect was observed in the 50 μg/ml POE treatment groups and was comparable to that with resveratrol (Figure 1B). There was no statistically significant difference in survival rate between the treatment groups (25 and 75 μg/ml) and the control group (Figure 1B). However, pretreatment with 50 μg/ml POE significantly improved the mean lifespan, median lifespan and maximum lifespan of worms under H_2_O_2_-induced oxidative stress (Supplementary Table S2; *p* < 0.05). Last, a similar result was also observed: the survival rate of POE (50 μg/ml) pretreated worms was higher than that of the control group under thermal stress conditions, although the effect was not as good as that in the resveratrol treatment group (Figure 1C; Supplementary Table S2). As expected, the 50 μg/ml POE treatment group exhibited the highest mean and maximum survival times, which were 11.2 ± 0.41 h and 20.50 ± 2.65 h, respectively (Supplementary Table S2; *p* < 0.05).

These results indicated that supplementation with POE has the potential to resist UV- and H_2_O_2_-mediated oxidative stress and 35°C-mediated heat stress in the *C. elegans* model.

### 3.3 Effect of *P. oxalate* extract on malondialdehyde content and antioxidant enzyme activity in *C. elegans*

**FIGURE 2:**
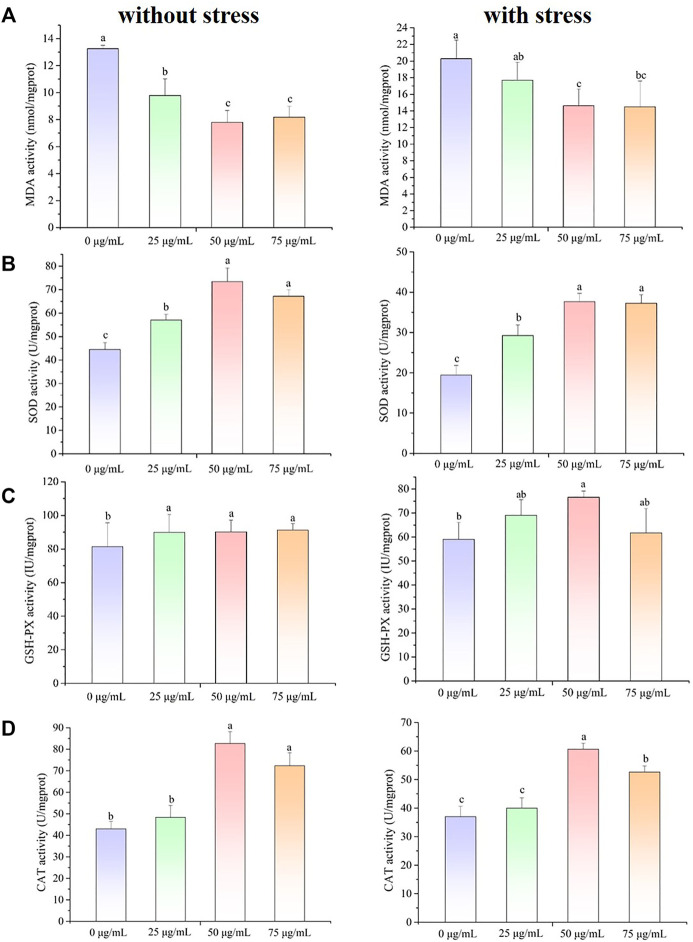
The effect of POE on the antioxidant defense system in *C. elegans* under normal and H_2_O_2_-induced oxidative stress conditions. **(A)** The MDA content. **(B)** The SOD activity. **(C)** The GSH-Px activity. **(D)** The CAT activity. Bars with no letters in common are significantly different (*p* < 0.05).

To elucidate the antioxidant and oxidative resistance properties of the POE *in vivo*, the MDA content and SOD and GSH-Px activities of POE-treated *C. elegans* were determined in H_2_O_2_-treated *C. elegans*, and the corresponding indexes were also determined under normal conditions. As shown in Figure 2A, the MDA content was decreased under both conditions compared to the control, indicating that POE was able to alleviate lipid peroxidation in *C. elegans* under normal and pressure conditions. Furthermore, since SOD and GSH-Px are the main ROS scavenging enzymes in the antioxidant defence system of *C. elegans*, we further measured the activities of antioxidant enzymes. As expected, compared with the control group, the SOD activity of *C. elegans* treated with POE was significantly increased under H_2_O_2_-induced oxidative stress conditions (Figure 2B; *p* < 0.05). A similar result was also observed in the absence of stress (Figure 2B). For GSH-Px and CAT activity, the enzyme activity in the POE treated group was significantly increased with and without pressure, compared with the control group (Figures 2C,D; *p* < 0.05). It was obvious that POE showed an excellent *in vivo* antioxidant capacity to activate the antioxidant defence system of *C. elegans*.

### 3.4 Effect of *P. oxalate* extract on Reactive oxygen species accumulation in *C. elegans*

**FIGURE 3:**
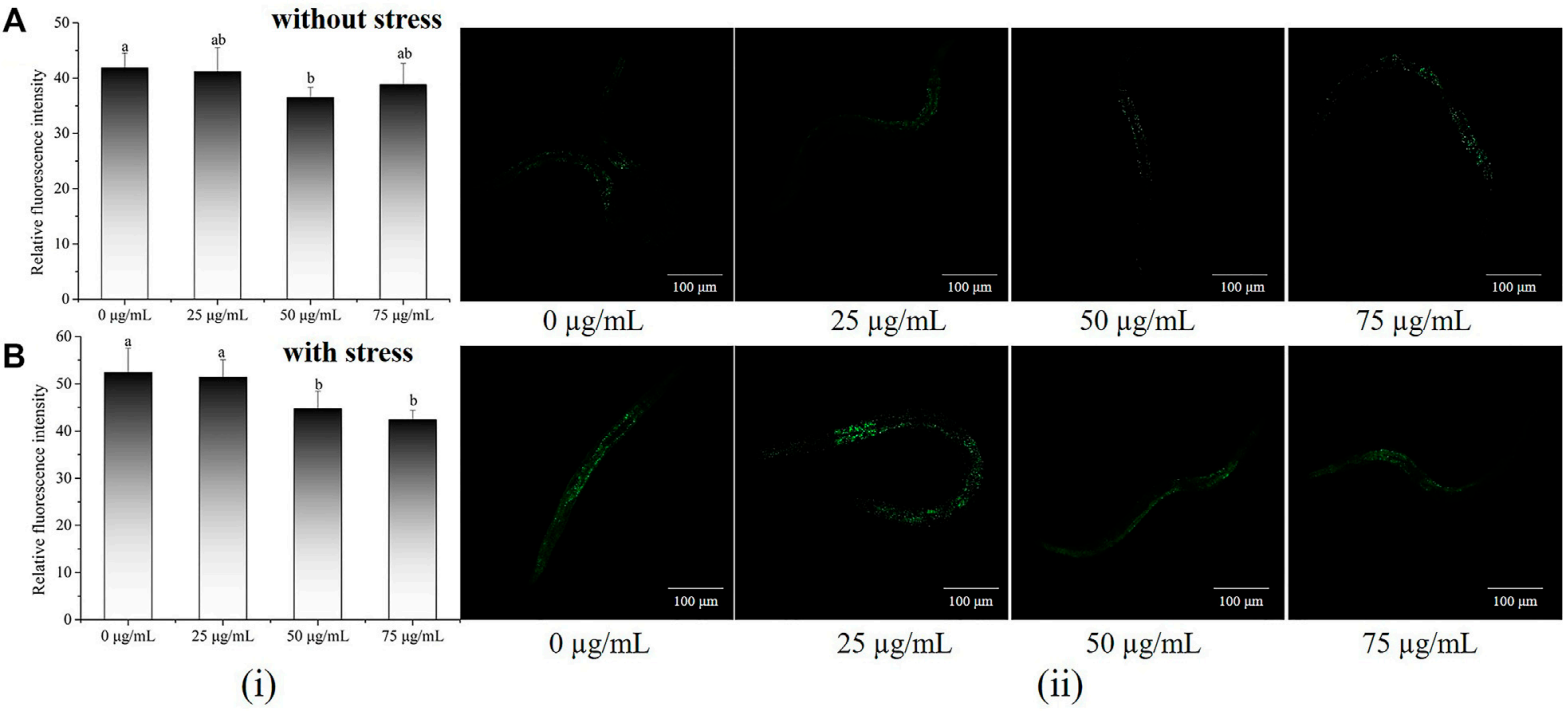
The effect of POE on intracellular levels of ROS in *C. elegans*. **(A)** Accumulation of ROS in *C. elegans* under normal conditions. **(B)** Accumulation of ROS in *C. elegans* under H_2_O_2_-induced oxidative stress. (i): Relative fluorescence intensity of worms was quantified using ImageJ software. (ii): Representative image of worms which was treated with POE in both conditions. Bars with no letters in common are significantly different (*p* < 0.05).

To further delve into the antioxidant potential of POE, the ROS levels of *C. elegans* were assessed under normal or stressful conditions. As shown in Figures 3A,B, higher ROS levels were found in worms with or without POE treatment under oxidative stress than in the absence of oxidative stress, indicating that H_2_O_2_ caused the accumulation of ROS in worms. Furthermore, POE treatment resulted in a decrease in ROS levels compared to the controls regardless of the conditions (Figure 3), which was directly proportional to the reduction in fluorescence. The ROS levels were significantly decreased in the 50 and 75 μg/ml POE-treated groups under oxidative stress and in the 50 μg/ml POE-treated group under normal conditions compared to controls (Figures 3A,B; *p* < 0.05). It was obvious that POE showed a significant antioxidant capacity and could scavenge intracellular ROS to a certain extent, which was consistent with reducing MDA and enhancing the activities of SOD and GSH-P_X_.

### 3.5 Effects of *P. oxalate* extract on body length and lipofuscin accumulation in *C. elegans*

**FIGURE 4:**
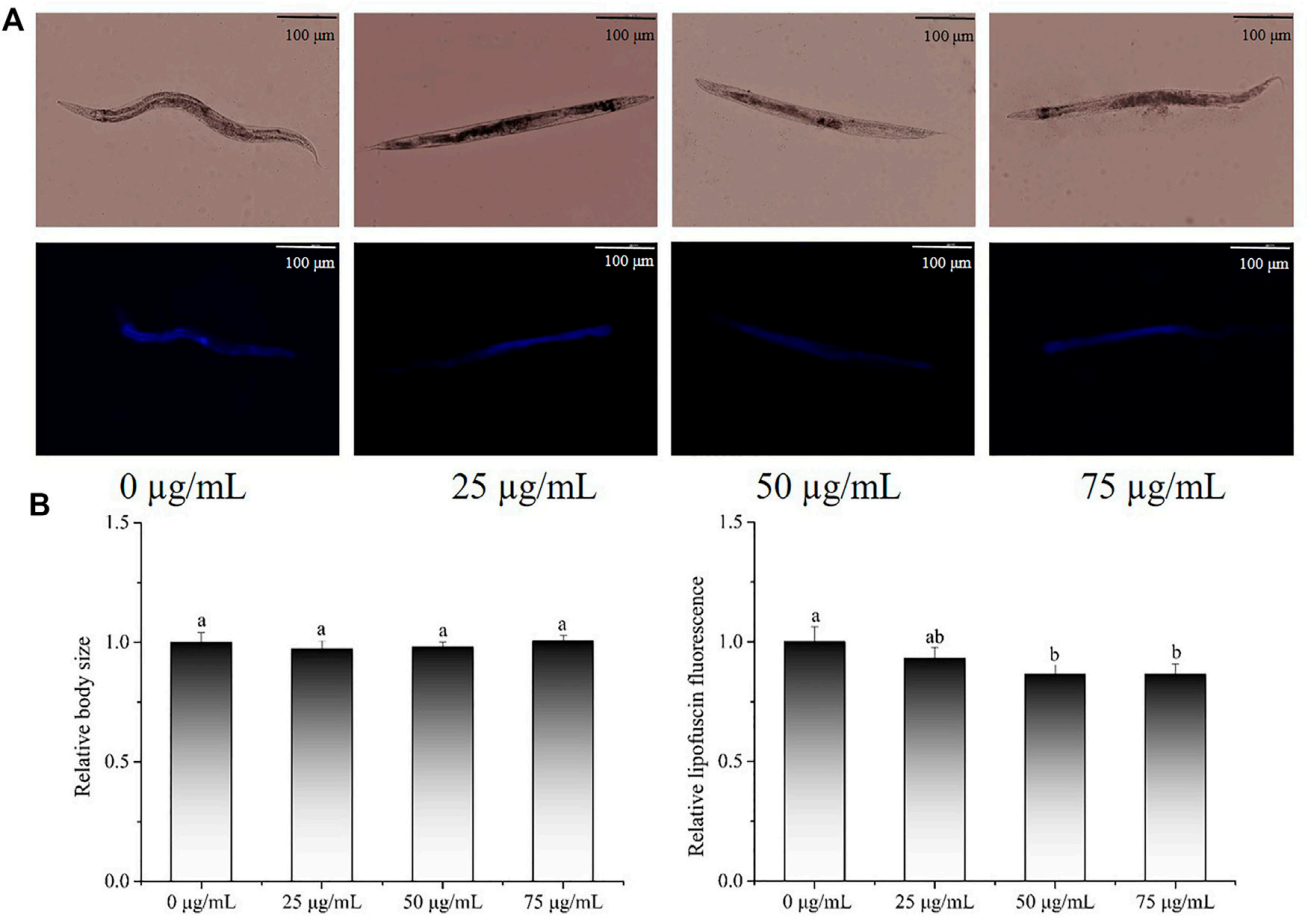
The effect of POE on lipofuscin accumulation and body size in *C. elegans*. **(A)** Representative images of fluorescence and bright field micrographs are shown, the scale bar was 100 μm; **(B)** body length and lipofuscin was measured and quantitated by ImageJ. Bars with no letters in common are significantly different (*p* < 0.05).

Lipofuscin, a marker of ageing, is commonly used to assess the health status of *C. elegans*. The body length and lipofuscin levels in *C. elegans* were also evaluated and representative images are presented in Figure 4A. In terms of body size (Figure 4B), there was no significant change between the POE treatment groups and the control group (*p* > 0.05), indicating that POE did not affect the body size of the worms. Relative fluorescence quantitative analysis showed that 50 and 75 µg/ml POE significantly reduced the accumulation of lipofuscin by 13.63% and 13.61%, respectively, in comparison with the control group (Figure 4B; *p* < 0.05).

### 3.6 Effect of *P. oxalate* extract on the lifespan of *C. elegans*

**FIGURE 5:**
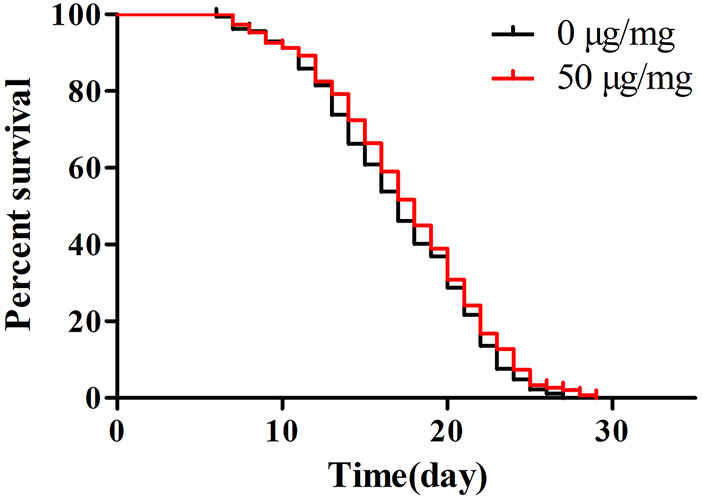
The effect of POE on lifespan of *C. elegans*. Results of lifespan experiments were analysed using the Kaplan-Meier survival model, and for significance by means of a long rank pairwise comparison test between the control and treatment groups. Differences compared to control group were considered significant at *p* < 0.05 (*), *p* < 0.01 (**) and *p* < 0.001 (***).

Next, we evaluated whether POE (50 µg/ml) could prolong the lifespan of worms. This concentration was chosen as the treatment dose because it was found to be more beneficial for reducing lipofuscin accumulation and enhancing stress tolerance. However, there was no significant difference in survival curves between the treatment and control groups (Figure 5; *p* > 0.05 by the log-rank test), indicating that although POE can alleviate the accumulation of age pigments, it is not sufficient to prolong the lifespan of worms.

### 3.7 Effect of *P. oxalate* extract on the fertility and movement of *C. elegans*

**FIGURE 6:**
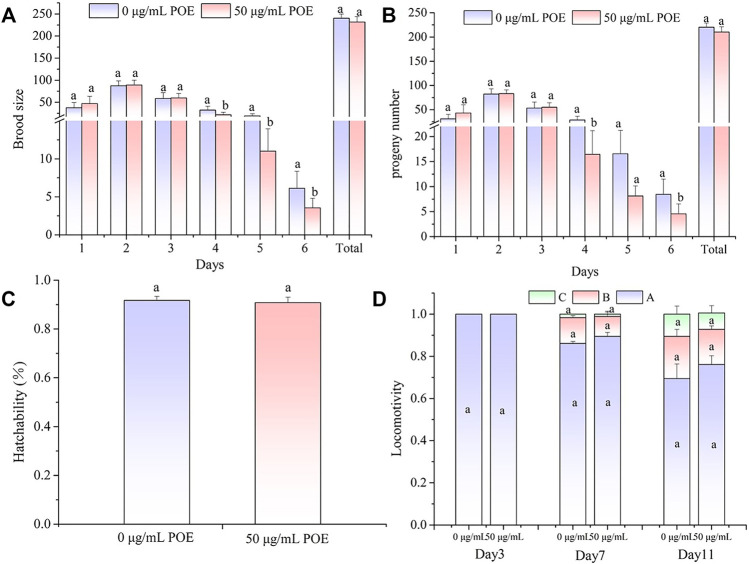
The effect of POE on reproduction and movement in *C. elegans*. **(A)** Brood size; **(B)** Progeny number; **(C)** Hatchability; **(D)** The three levels of locomotivity were measured and the individuals were classified according to the movement: A-free movement, B-movement after prodding, C-weak movement after prodding. Data were expressed as the mean ± SD (*n* = 3). Bars with different letters indicated statistical significance (*p* < 0.05).

Fertility and movement assays were performed to examine whether POE had some side effects on the physiological function of this dose (50 µg/ml). Analyses of fertility showed that the size of the brood and progeny number from Day 4 were slightly decreased after POE treatment, but there were no differences in total brood size, total progeny number or total hatchability in worms treated with POE when compared with the controls (Figures 6A–C).

In addition, we found that, as the worms aged, their motility gradually declined, and B-class and C-class locomotion began to appear in the middle and middle-late stages of the life cycle (Figure 6D). However, the movement assay did not show significant differences in locomotion ability between the POE-treated group and control group in the different stages of the life cycle (Figure 6D).

Considered together, these results showed that POE had no obvious effects on the reproductive and motor systems of *C. elegans*.

### 3.8 *P. oxalate* extract enhanced stress resistance in *C. elegans* by activating oxidative stress-inducible genes that might not be associated with *skn-1* but might be dependent on *daf-16* and *hsf-1*

**FIGURE 7:**
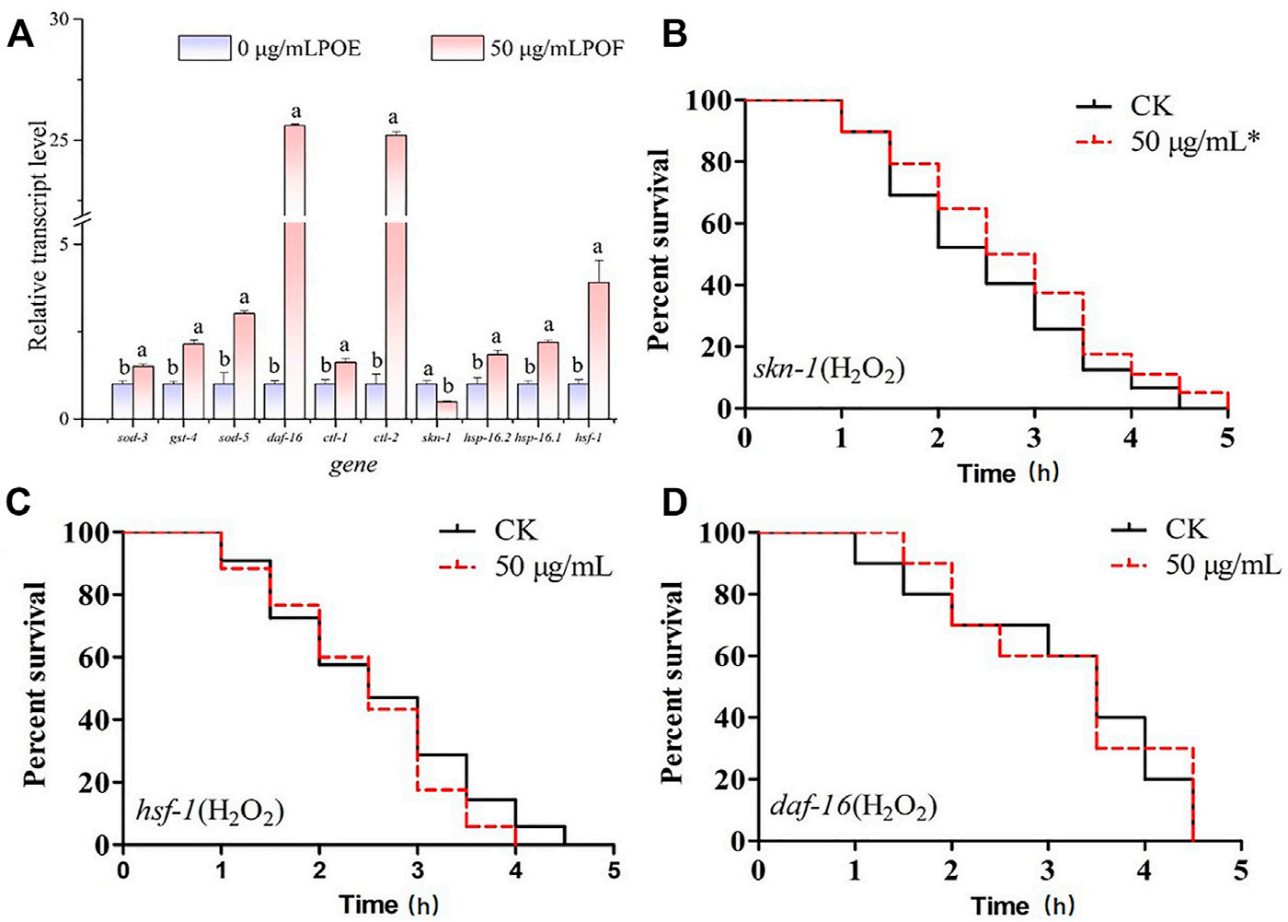
The molecular mechanism of POE in the antioxidant stress. **(A)** The expression of stress-related genes in *C. elegans* under H_2_O_2_-induced oxidative stress conditions. **(B)** The survival curve of *skn-1* mutant worms under H_2_O_2_-induced oxidative stress. **(C)** The survival curve of *hsf-1* mutant worms under H_2_O_2_-induced oxidative stress. **(D)** The survival curve of *daf-16* mutant worms under H_2_O_2_-induced oxidative stress. Data were expressed as the mean ± SD (*n* = 3). Bars with different letters indicated statistical significance (*p* < 0.05). * Significant *p*-value <0.05 by the log-rank test.

The antistress ability of POE has been proved, but the underlying molecular mechanisms require further study. Since POE can enhance antioxidant enzyme activity and reduce ROS accumulation, we further investigated the relative expression levels of oxidative stress-inducible genes (*sod-3*, *sod-5*, *gst-4*, *ctl-1*, and *ctl-2*) using RT‒qPCR. As indicated in Figure 7, the relative expression levels of various oxidative stress-inducible genes of in the POE-treated group were significantly higher than that of the control group, especially *ctl-2* (exhibiting a 25.2-fold increase). Moreover, it was observed that the relative expression levels of *daf-16* (25.59-fold), *hsf-1* (4.21-fold), *hsp-16.1* (2.19-fold) and *hsp-16.2* (1.82-fold) were upregulated significantly (*p* < 0.05) compared to the control group. However, the relative expression levels of *skn-1* were decreased 0.48 times. In addition, to further confirm the underlying molecular mechanisms, the *daf-16*, *skn-1*, and *hsf-1* mutants were used to evaluate the effects of POE on lifespan in *C. elegans* mutants under H_2_O_2_-induced oxidative stress. We found that the survival rate of POE-treated *skn-1* mutants was significantly increased compared to that of the control group (Figure 7B, *p* < 0.05), confirming that POE might act independently of *skn-1*. However, the *daf-16* and *hsf-1* mutants did not show a protective effect of POE on worm lifespan (Figures 7C,D; *p* > 0.05), indicating that *daf-16* and *hsf-1* might be necessary for POE to improve stress resistance.

### 3.9 Effect of *P. oxalate* extract on DNA damage protective activity

#### 3.9.1 DNA nicking assay for hydroxyl radical scavenging activity

**FIGURE 8:**
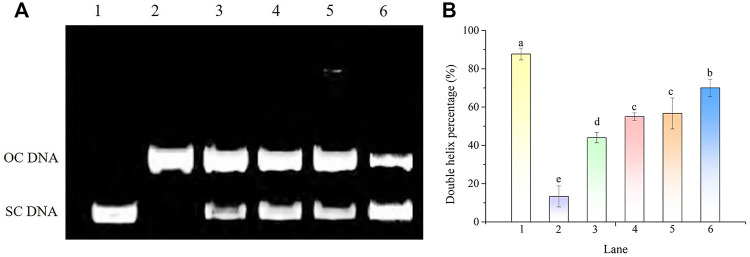
The DNA protective effect of POE against •OH generated by Fenton’s reagent. **(A)** Electrophoretogram. Lanes 1 and 2 were the normal DNA treated with and without 1 mM FeSO_4_ and 1 mM H_2_O_2_, respectively. Lanes 3–6 were treated with various concentrations of POE (25, 50, and 75 μg/ml) and VE (500 μM). **(B)** Double helix percentage. Bars with different letters indicated statistical significance (*p* < 0.05).

The protective effect of POE on hydroxyl radical-induced DNA oxidative damage is shown in Figure 8. The plasmid DNA corresponding to the prominent faster moving band was the supercoiled form (SC DNA) (Figure 8A, **Lane 1**). After the addition of Fe^2+^ and H_2_O_2_, the supercoiled circular DNA completely converted into the open circular or linear forms (OC DNA) referred to as the slowest moving line (Figure 8A, **Lane 2**) and the DNA double helix percentage was 13% (Figure 8B), suggesting that the hydroxyl radicals generated by the Fenton reaction damaged the original structure of DNA and led to DNA nicking. However, when different concentrations of POE were added, part of the OC DNA reverted to SC DNA (Figure 8A, **Lanes 3–5**) and their DNA double helix percentages were 44%, 55%, and 57%, respectively (Figure 8B), indicating that POE can effectively relieve hydroxyl radical-induced DNA damage.

#### 3.9.2 Cytochalasin blocked micronucleus assay in lymphocytes

**FIGURE 9:**
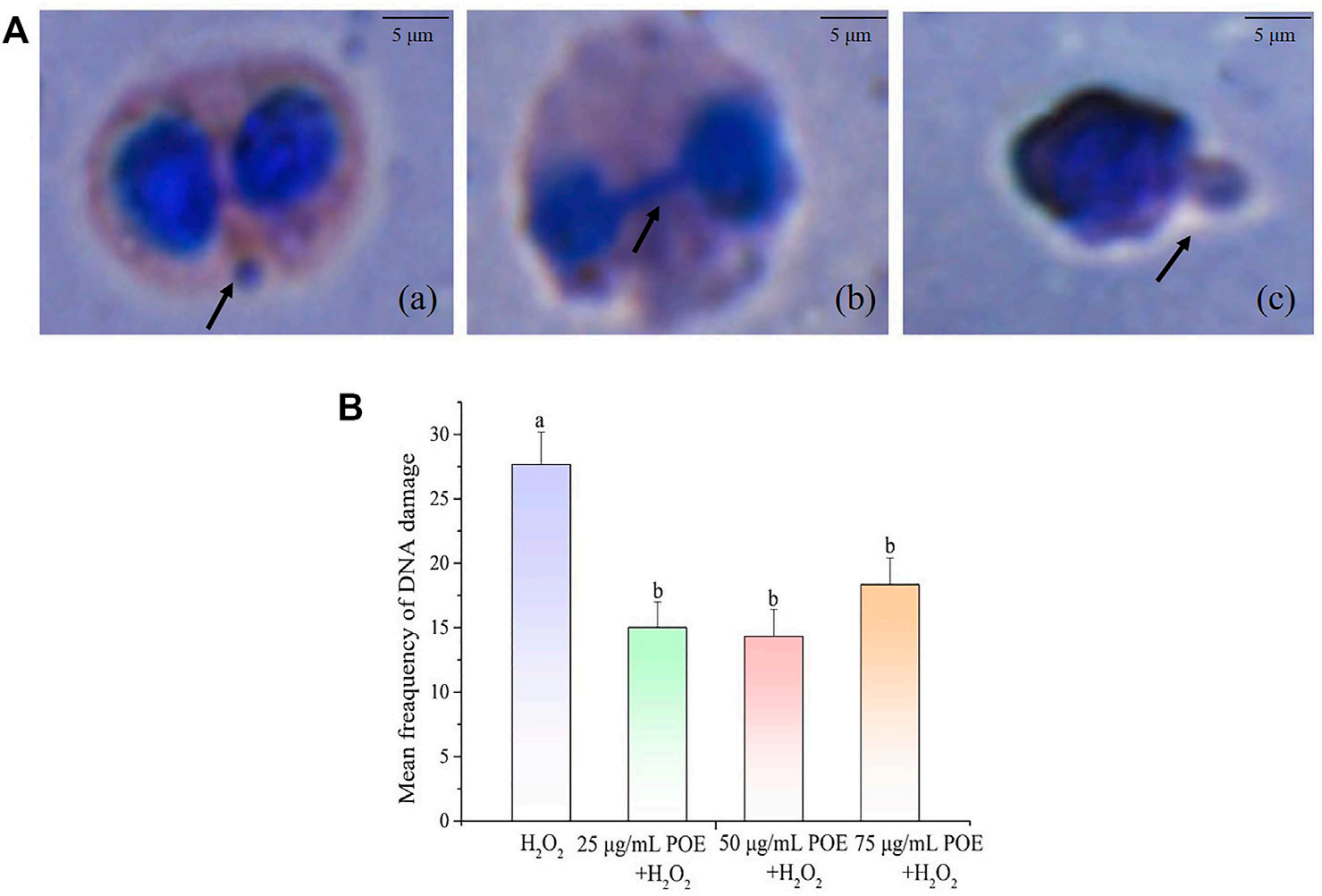
The effect of POE on DNA damage protection in lymphocyte. **(A)** Various forms of DNA damage seen as (a) MN, (b) nucleoplasmic ridge and (c) nuclear bud on cells treated with H_2_O_2_, the scale bar was 5 μm. **(B)** The mean frequency of DNA damage in human lymphocytes exposed to H_2_O_2_ (250 µM), H_2_O_2_ (250 µM) + POE (25, 50, and 75 μg/ml). Data were expressed as the mean ± SD (*n* = 3). Bars with different letters indicated statistical significance (*p* < 0.05).

The DNA damage protection of POE was also investigated using the CBMN assay (Figure 9). As shown in Figure 9A, the forms of DNA damage were MN, nuclear buds and nucleoplasmic bridges and the mean frequency of DNA damage in the 25, 50, and 75 µg/ml treatment groups was 15 ± 2, 14.3 ± 2 and 18 ± 2, respectively, exhibiting a significant decrease in micronucleus frequency compared with the controls (27.7 ± 2.5) (Figure 9B; *p* < 0.05). The current study revealed that POE was able to improve the protection against DNA damage in lymphocytes.

## 4 Discussion

Endophytic fungi can produce medicinal ingredients with the same or similar functions as the host plant by long-term mutualism with host plants (Aly et al., 2011). *L. chuanxiong* Hort (Umbelliferae), a medicinal and edible plant, is commonly used for the promotion of good body health, anti-inflammation, antioxidation, neuron protection and blood vessel elasticity (Yuan et al., 2020). It has also been reported that the endophytic fungus *L. chuanxiong* can produce abundant secondary metabolites that might be applied for various purposes (Li et al., 2020; Cao et al., 2021). In our earlier study, *P. oxalate* isolated from *L. chuanxiong* exhibited strong antioxidant activity in chemical-based assays and *P. oxalate* extracts (POE) contained rich polyphenols such as ferulic acid, hesperidin and chlorogenic acid (Tang et al., 2021). In the present study, the biological activities of POE were further studied with regard to DNA damage protection effects and stress resistance properties.

Oxidative stress is closely related to the pathogenesis of various diseases such as cancer and neurological diseases (Xie et al., 2013). According to our results, we first found that POE could improve tolerance against oxidative stress (UV, 35°C and H_2_O_2_-induced) in *C. elegans* and the protection was not given in a concentration-dependent manner. In fact, only treatment with POE at 50 μg/ml significantly improved the worm’s ability to respond to various stressors, while 75 μg/ml POE showed no effect.

The reason for this finding could be that POE has been proven to contain complex chemical components, while some compounds, such as caffeic acid, exhibit a hormetic response, eventually producing a deleterious effect when its content increases to greater than certain levels (Pietsch et al., 2011; Gutierrez-Zetina et al., 2021). Therefore, we hypothesized that POE concentrations of 50–75 μg/ml could represent the inflection point from which the beneficial effects induced by the POE in *C. elegans* would begin to decline. Furthermore, some authors have also observed that the survival of oxidative stress-induced *C. elegans* was significantly improved with increasing extract concentrations within a certain level, but decreased at higher concentrations (Dueñas et al., 2013; Duangjan et al., 2019b), which is consistent with our results. Notably, moderate-dose POE was found to enhance the mean lifespan of *C. elegans* under H_2_O_2_-mediated oxidative stress (increased by 17.12%), comparable to resveratrol (positive control) and some crude extracts such as polysaccharides (Lin et al., 2020). Moreover, ferulic acid, hesperidin, chlorogenic acid and other polyphenols with antioxidant activity have been reported (Gülçin, 2012; Li and Schluesener, 2017), while their content is very low in POE. However, some researchers have attributed the biological effect of the extract to the synergic and additive action among multiple chemicals (Vayndorf et al., 2013; Wang et al., 2018). Therefore, we proposed that the outstanding stress resistance activity of POE might be attributed to the interactions among various compounds of endophytic fungi instead of single secondary metabolites. However, it is inevitable that the study also had some limitations, such as lack of studies on biological effects of other single components of POE, requiringes further study in the future.

**FIGURE 10:**
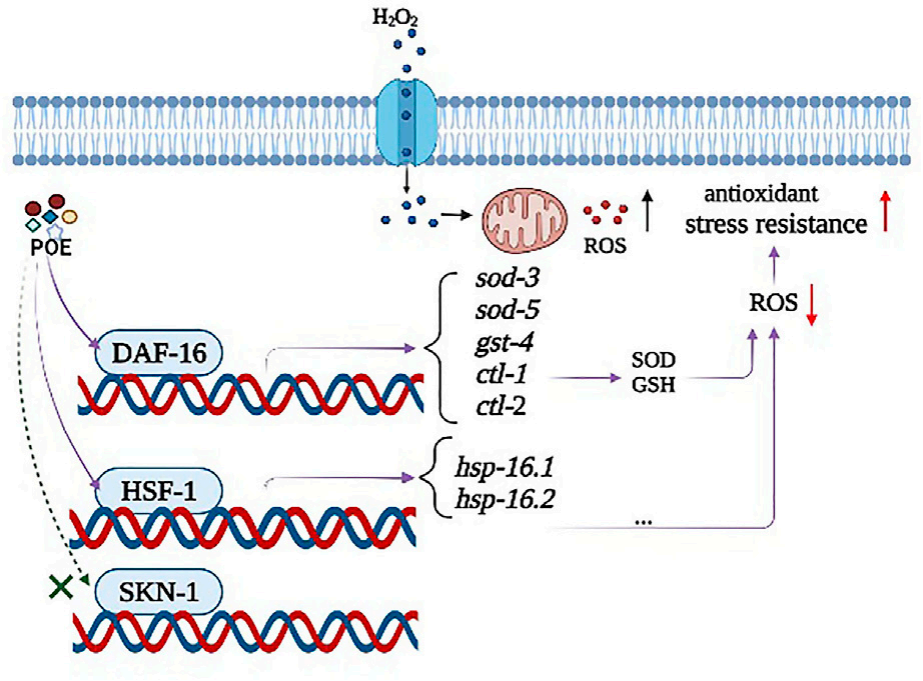
A possible model of the mechanism of action of POE-mediated stress resistance in *C. elegans*. POE alleviates the accumulation of ROS by activating the antioxidant defense system, and ultimately improves the anti-stress ability of *C. elegans*. The observed effects were mediated, at least in part, by the two master regulators DAF-16 and HSF-1 signaling pathways rather than SKN-1.

It is widely believed that compounds exert their biological effects not only because of their role as conventional antioxidants but also because of their ability to modulate the expression of related genes and act simultaneously on complex signalling pathways (Mansuri et al., 2014). In *C. elegans*, the *daf-16* gene encodes the transcription factor DAF-16, which is considered to be a crucial regulator in the insulin/IGF-1 signalling pathway and regulates stress-related gene expression in cells (Sen et al., 2020). Thus, extracts can increase the ability to prevent or repair stress damage in *C. elegans* by activating the daf-16 transcription factor and reducing IIS pathway activity (Ayuda-Durán et al., 2019). Moreover, *sod-3,4*, *ctl-1*, *ctl-2*, and *gst-4* are target genes of DAF-16, which encode proteins responsible for antioxidant defences (Murphy et al., 2003). In our study, the survival curve of the *daf-16* mutant showed no significant change after POE treatment, suggesting that the observed overexpression of these genes following treatment with POE could be related to the increase in the expression of DAF-16 (Gutierrez-Zetina et al., 2021). Therefore, POE improved the stress resistance of *C. elegans* by activating the daf-16 transcription factor, further promoting the expression of downstream target genes. Moreover, some antioxidant enzymes can reduce or eliminate excess free radicals in the body through biochemical reactions to maintain body stability. For example, superoxide dismutase-3 (SOD-3) catalyses the conversion of superoxide radicals to hydrogen peroxide and diatomic oxygen (Moreno-arriola et al., 2014). Thus, the overexpression of these target genes in *C. elegans* can also explain the decreased ROS level in the present study. In addition to DAF-16 signalling, SKN-1 is also an important regulator of oxidative stress resistance, mobilizing a conservative phase 2 detoxification response and promoting the activation of multiple genes in *C. elegans* (Tullet et al., 2008). However, there was no significant change in the expression of the *skn-1* gene in worms treated with POE, and POE treatment significantly increased the longevity of the *skn-1* mutant, indicating that it might not be conducive to POE-mediated resistance. In addition, another important gene was *hsf-1*. This gene encodes the thermal shock transcription factor HSF-1 which regulates the expression of various molecular chaperones (HSP-16.1 and HSP-16.2) to defend against thermal or oxidative stress (Hsu et al., 2003; Hsu et al., 2003; Kumsta et al., 2017). In the present study, overexpression of *hsf-1*, *hsp-16.1*, and *hsp-16.2* and the lack of effect of POE on the longevity of *hsf-1* mutants provide evidence that the HSF-1 pathway might be necessary for the antistress properties of POE. Accordingly, we hypothesized that the mechanism by which POE improves the stress resistance of *C. elegans* is that POE activates daf-16 to activate oxidative stress-inducible genes (*sod-3, sod-5*, *gst-4*, *ctl-1*, and *ctl-2*) and hsf-1 to promote the expression of downstream heat stress-inducible genes (*hsp-16.1* and *hsp-16.2*), rather than *skn-1* under stress conditions (Figure 10).

In general, an increase in lifespan is often accompanied by an increase in stress resistance in *C. elegans* (Murphy et al., 2003). Lipofuscin is an autofluorescent compound that accumulates gradually with the ageing of *C. elegans* (Clokey and Jacobson, 1986). However, our results showed that POE decreased lipofuscin levels in worms, while it did not extend life, indicating that POE showed some health benefits, but they were not sufficient to prolong the lifespan of worms because the lifespan is affected by multiple factors. Thus, the enhanced antistress ability of worms treated with POE found in the present study is not in line with lifespan extension, consistent with the observations of Duangjan et al. (2021) working. Moreover, lifespan analysis was used to evaluate the long-term toxicity of extracts (Romero-Márquez et al., 2022). Thus, the results also showed that POE has no long-term toxicity to worms. Ideally, there should be no harm to health indicators while showing some beneficial biological effects. However, some studies have measured only one or two indicators to evaluate the side effects of extracts on worms (Moliner et al., 2020; Duangjan et al., 2021). Therefore, this study comprehensively evaluated from four perspectives: long-term and short-term acute toxicity, reproduction, locomotion and growth. It demonstrated that POE has a beneficial effect on enhancing stress resistance without adverse effects.

There is a considerable amount of evidence revealing a correlation between DNA lesions and the occurrence of chronic and degenerative illness (Thanan et al., 2014); for example, DNA strand breaks caused by hydroxyl radical- induced persistent oxidative damage are suspected to be a major cause of carcinogenesis (Powell et al., 2005; Chandrasekara and Shahidi, 2011). In our study, the DNA damage protection capacity of POE was evaluated on pBR322 plasmid DNA when treated with Fenton’s reagent. The hydroxyl radical generated by the Fenton reactant can attack DNA and cause a dramatic scission of the supercoiled (SC) DNA strand to open circular (OC) strands (Qian et al., 2008). Under such conditions, POE can may interfere with the reaction of Fe^2+^with H_2_O_2_ or directly quench hydroxyl radicals by providing an electron due to its high antioxidant potential and further protect the supercoiled plasmid DNA against hydroxyl radicals (Chandrasekara and Shahidi, 2011). Moreover, a significant reduction in CBMN was observed in H_2_O_2_ treated lymphocytes when exposed to POE, which again demonstrated its antioxidant and DNA damage protection effects. This result is in line with previous studies in which *Aspergillus fumigatus* (Kaur et al., 2021) and *P. oxalicum* (Kaur et al., 2020) extracts showed DNA damage protection. Abundant phenolic compounds, viz; hesperetin, ferulic acid, alternariol and apigenin have been found in POE in our previous studies (Tang et al., 2021), which might be responsible for its biological effects. For example, hesperidin not only showed strong free radical scavenging ability *in vitro* (Wilmsen et al., 2005), but it also provided strong cellular antioxidant protection to alleviate oxidative stress and DNA damage (Sahu et al., 2013). Therefore, although the underlying mechanisms of DNA damage protection are not fully understood, the protective ability of POE could be related to the abundant secondary metabolites of *P. oxalate*.

## 5 Conclusion

In this study, the antioxidant activity and stress resistance of POE were investigated in *C. elegans*, and the protection activity against DNA damage of POE was evaluated by the pBR322 plasmid and lymphocytes. Our study revealed that POE might effectively counteract UV, 35°C and H_2_O_2_-induced oxidative stress without compromising the growth, reproduction and locomotion of *C. elegans*. The partial oxidative resistance properties of POE can be attributed to diminished intracellular ROS, as well as elevated activity of antioxidant enzymes (SOD CAT and GSH-P_X_). The possible mechanism by which POE enhances stress resistance in *C. elegans* is mediated by activating the DAF-16 and HSF-1 pathways and promoting the overexpression of stress response genes. In addition, we found that POE had a protective effect against Fenton reaction produced DNA nicking and H_2_O_2_-induced DNA damage in lymphocytes. In summary, this study is the first to report the antistress effects and DNA damage protection potential of endophytic fungus *P. oxalate* extracts, which could be a potential resource for treating oxidative stress and DNA damage diseases. However, the underlying mechanisms of the biological effects and more *in vivo* interventions with complex model organisms are needed to support the therapeutic potential of POE in the future.

## Data Availability

The original contributions presented in the study are included in the article/Supplementary Material, further inquiries can be directed to the corresponding authors.

## Abbreviations

Res, resveratrol; VE, vitamin E; MDA, malondialdehyde; SOD, superoxide dismutase; CAT, catalase; GSH-Px, glutathione peroxidase; ROS, reactive oxygen species; POE, *Penicillium oxalate* extract.
