# Supplementary material for: The endophytic fungus Penicillium oxalicum isolated from Ligusticum chuanxiong Hort possesses DNA damage-protecting potential and increases stress resistance properties in Caenorhabditis elegans
Source: Front Pharmacol. 2022 Aug 30;13:983716. doi: 10.3389/fphar.2022.983716 (PMC9468742; doi:10.3389/fphar.2022.983716)
Supplement: Supplementary file 1 [file DataSheet1.docx]

**Table S1** Primer sequences for qRT-PCR analysis

| Gene | Primer |
| --- | --- |
| *hsp-16.1* | CTGAATCTTCTGAGATTGTTAAC (F)  TTTGTTCAACGGGCGCTTGC (R) |
| *hsp-16.2* | CTGCAGAATCTCTCCATCTGAGTC (F)  AGATTCGAAGCAACTGCACC (R) |
| *hsf-1* | GAAATGTTTTGCCGCATTTT (F) |
|  | CCTTGGGACAGTGGAGTCAT (R) |
| *sod-3* | CCAACCAGCGCTGAAATTCAATGG (F)  GGAACCGAAGTCGCGCTTAATAGT (R) |
| *sod-5* | GAACTGCTGTCTTCGGAACTG (F)  CCATGAAGTCCTGGTGACAAT (R) |
| *gst-4* | ATGCTCGTGCTCTTGCTGAG(F)  GACTGACCGAATTGTTCTCCAT(R) |
| *skn-1* | CACGCCGTCAGCGAAGTA(F)  ATGCTCGGTGAGTATTGG(R) |
| *daf-16* | CTTCAAGCCAATGCCACTACC (F)  GGAGATGAGTTGGATGTTGATAGC (R) |
| *ctl-1* | GCGGATACCGTACTCGTGAT (F)  GTGGCTGCTCGTAGTTGTGA (R) |
| *ctl-2* | GAGAATGTGCCAGAACTTTGC (F)  CTTGACACGAGCTCCAAAATC (R) |
| *act-1* | TCCAAGAGAGGTATCCTTAC (F)  CGGTTAGCCTTTGGATTGAG (R) |

**Table S2** Statistical analysis of survival time of *C. elegans*.

| Stressors | Group | Mean time^(1)^ | Median time^(2)^ | Median time^(3)^ | P-Value^(4)^ |
| --- | --- | --- | --- | --- | --- |
| Ultraviolet | Control | 4.63±0.17_c_ | 3.24±0.20_c_ | 6.17±0.47_b_ |  |
|  | POE(25 μg/mL) | 4.93±0.12_bc_ | 3.62±0.09_bc_ | 6.83±0.47_ab_ | 0.20 |
|  | POE(50 μg/mL) | 5.28±0.18_ab_ | 3.97±0.20_b_ | 7.17±0.47_ab_ | <0.05 |
|  | POE(75 μg/mL) | 4.95±0.37_bc_ | 3.61±0.40_bc_ | 7.17±0.47_ab_ | 0.15 |
|  | Res(22.5 μg/mL) | 5.58±0.44_a_ | 5.33± 0.58_a_ | 8±1_a_ | <0.01 |
| H_2_O_2_ | Control | 2.16±0.20_b_ | 1.80±0.14_b_ | 3.62±0.22_b_ |  |
|  | POE(25 μg/mL) | 2.05±0.38_b_ | 1.85±0.14_b_ | 4.08±0.29_b_ | 0.31 |
|  | POE(50 μg/mL) | 2.53±0.05_a_ | 2.13±0.04_a_ | 4.58±0.29_a_ | <0.01 |
|  | POE(75 μg/mL) | 2.21±0.07_ab_ | 1.90±0.02_b_ | 4.42±0.29_a_ | 0.50 |
|  | Res(22.5 μg/mL) | 2.61±0.12_a_ | 2.33±0.29_a_ | 4.87±0.28_a_ | <0.01 |
| Heat | Control | 9.43±0.18_c_ | 8.72±0.30_c_ | 17.83±0.58_c_ |  |
|  | POE(25 μg/mL) | 10.35±0.20_b_ | 9.63±0.36_b_ | 18.83±1.53_bc_ | 0.10 |
|  | POE(50 μg/mL) | 11.2±0.41_b_ | 10.74±0.29_ab_ | 20.50±2.65_b_ | <0.01 |
|  | POE(75 μg/mL) | 9.87±0.05_bc_ | 9.58±0.27_bc_ | 18.50±0.82_bc_ | 0.14 |
|  | Res(22.5 μg/mL) | 12.87±0.12_a_ | 11.56±0.21_a_ | 22.50±1.68_a_ | <0.001 |

All data are presented as the mean ± SD, and different letters in columns indicate that the values are significantly different (р < 0.05).

(1) Mean survival time: MLS =1/n∑_j_ (Xj+X_j+1_)/2d_j_, where j is the age category , d_j_ is the number of worms that died in the age interval (x_j_, x_j+1_), and n is the total number of worms.

(2) The median lifespan is the time at which fraction survival equals 50%.

(3) The maximum lifespan is the time at which fraction survival equals 0%.

(4) P-value was calculated using the log-rank test by comparing the POE-treated group with control.
